# Development of Silk Fibroin-Based Non-Crosslinking Thermosensitive Bioinks for 3D Bioprinting

**DOI:** 10.3390/polym15173567

**Published:** 2023-08-28

**Authors:** Juo Lee, Sangbae Park, Sungmin Lee, Hae Yong Kweon, You-Young Jo, Jungsil Kim, Jong Hoon Chung, Hoon Seonwoo

**Affiliations:** 1 Department of Animal Science & Technology, Sunchon National University, Suncheon 57922, Republic of Korea; juolee23@naver.com; 2 Interdisciplinary Program in IT-Bio Convergence System, Sunchon National University, Suncheon 57922, Republic of Korea; 3 Department of Convergence Biosystems Engineering, Chonnam National University, Gwangju 61186, Republic of Korea; sb92park@gmail.com; 4 Department of Rural and Biosystems Engineering, Chonnam National University, Gwangju 61186, Republic of Korea; 5 Interdisciplinary Program in IT-Bio Convergence System, Chonnam National University, Gwangju 61186, Republic of Korea; 6 Department of Mechanical Engineering, Sunchon National University, Suncheon 57922, Republic of Korea; leecm1009@naver.com; 7 Division of Industrial Insect and Sericulture, National Institute of Agricultural Sciences, Rural Development Administration, Wanju 55365, Republic of Korea; hykweon@korea.kr; 8 Department of Agricultural Biology, Apiculture Division, National Institute of Agricultural Sciences, Rural Development Administration, Wanju 55365, Republic of Korea; yyjo@korea.kr; 9 Department of Bio-Industrial Machinery Engineering, Kyungpook National University, 80 Daehakro, Bukgu, Daegu 41566, Republic of Korea; jungsil.kim@knu.ac.kr; 10 ELBIO Inc., Seoul 08812, Republic of Korea; 11 Department of Convergent Biosystems Engineering, College of Life Science and Natural Resources, Sunchon National University, Suncheon 57922, Republic of Korea

**Keywords:** silk fibroin, bioprinting, bioink

## Abstract

Three-dimensional (3D) bioprinting holds great promise for tissue engineering, allowing cells to thrive in a 3D environment. However, the applicability of natural polymers such as silk fibroin (SF) in 3D bioprinting faces hurdles due to limited mechanical strength and printability. SF, derived from the silkworm Bombyx mori, is emerging as a potential bioink due to its inherent physical gelling properties. However, research on inducing thermosensitive behavior in SF-based bioinks and tailoring their mechanical properties to specific tissue requirements is notably lacking. This study addresses these gaps through the development of silk fibroin-based thermosensitive bioinks (SF-TPBs). Precise modulation of gelation time and mechanical robustness is achieved by manipulating glycerol content without recourse to cross-linkers. Chemical analysis confirms β-sheet conformation in SF-TPBs independent of glycerol concentration. Increased glycerol content improves gelation kinetics and results in rheological properties suitable for 3D printing. Overall, SF-TPBs offer promising prospects for realizing the potential of 3D bioprinting using natural polymers.

## 1. Introduction

The goal of regenerative medicine is to regenerate or replace damaged or necrotic tissues with functional tissues inherent to the tissue type [1]. To achieve this, tissue engineering requires a comprehensive understanding of the biological knowledge related to the proliferation and differentiation of cells within the context of biomaterials [2,3]. Given the three-dimensional nature of our bodies, one of the fundamental challenges in tissue engineering has been the cultivation of cells on 3D scaffolds. Conventional techniques used in scaffold fabrication include solvent casting, particulate leaching, gas foaming, fiber mesh/fiber bonding, phase separation, melt molding, emulsion freeze-drying, solution casting, and freeze-drying, among others. However, these existing methods often have limitations in achieving precise pore sizes [3,4,5], pore shapes, high interconnectivity, and mechanical strength. Such conventional approaches often involve manually seeding cells, which can result in inconsistent cell incorporation, posing issues when precise cell alignment is required based on tissue demands and functions. For instance, achieving consistent alignment of endothelial cells to form blood vessels or arranging osteogenic cells to form mineralized clusters can be challenging. To overcome these limitations, advanced technologies such as 3D printing have been explored in research [1]. Ultimately, this can lead to the production of matrix scaffolds that can more effectively promote the regeneration of functional tissues.

Three-dimensional bioprinting technology has the potential to address several challenges that have inhibited progress in tissue engineering applications, such as forming complex structures for tissue-specific needs, maintaining mass nutrient delivery in scaffolds, and subsequent tissue processing [6]. This is important for modulating cell behavior under in vivo conditions by reproducing interactions between cells and the extracellular matrix (ECM) that are not seen in normal two-dimensional cell culture [7]. Currently, a significant challenge in 3D culture is the inadequate supply of nutrients and oxygen to the core of the structure. To address this issue, research focusing on 3D bioprinting in mesh-like structures is underway. Furthermore, in pursuit of more comprehensive solutions, there have been reported instances of studies involving the integration of capsules that can provide oxygen and nutrients until neovascularization occurs [8]. Bioprinting exhibits the capacity to replicate intricate tissue architectures, as documented in the literature. Its prowess lies in aligning cells and provoking favorable cellular responses through interactions with biomaterials. The advancement of technology in developing and regenerating complex tissue structures through such approaches is progressing steadily. The most commonly used bioprinting technologies include extrusion, particle fusion, photo-irradiation, and inkjet-based bioprinting [9,10]. Among them, extrusion-based bioprinting is one of the most popular technologies due to its compatibility with various bioinks, ease of operation, and relatively low cost [11,12]. An important technology for 3D bioprinting is the production of bioinks [13]. An ideal bioink should support cell and tissue growth (cell adhesion and differentiation), adequate mechanical strength (sol–gel transition and self-standing), and mass metabolism (nutrient and growth factor transfer). And it must have a function similar to the minimal ECM [14].

The field of 3D bioprinting utilizes various types of bioinks, which can be broadly categorized into synthetic polymers and naturally derived polymers [15]. Synthetic polymers are man-made materials that offer precise control over their chemical composition and physical properties [16]. Synthetic polymer bioinks commonly used in 3D bioprinting include polyethylene glycol (PEG) [17], polycaprolactone (PCL) [18], poly(lactic-co-glycolic acid) (PLGA) [19], and polyvinyl alcohol (PVA) [20]. These polymers offer customizability in terms of mechanical properties, degradation rates, and functional groups for biofunctionalization. They can be precisely engineered to mimic the extracellular matrix (ECM) and provide structural support for cells. However, synthetic polymers may lack inherent bioactivity and cell recognition motifs, requiring additional modifications to enhance interactions between cells and materials [21]. On the other hand, naturally occurring polymers are derived from biological sources such as plants, animals, or microorganisms. These polymers often possess inherent bioactivity and biocompatibility, making them suitable for tissue engineering and regenerative medicine applications [22]. Examples of naturally derived polymeric bioinks include alginate [23], gelatin [24], collagen [25], fibroin [26], chitosan [27], and hyaluronic acid [28]. Naturally derived polymers often possess intrinsic bioactivity and cell-adhesive properties that promote cell function and tissue regeneration. They can provide a more biomimetic environment for cells, allowing for better cell attachment, proliferation, and differentiation. However, naturally derived polymers can have limited mechanical strength and stability, requiring additional cross-linking or reinforcement strategies for 3D bioprinting [29]. Hence, a silk-based hydrogel employing a physical crosslinking approach has emerged, and research on its utilization in 3D bioprinting has been documented.

Naturally derived proteins from silk fibers can be processed into diverse biomaterials, including 3D sponges, films, nanofibers, nanoparticles, and microparticles. Silk is characterized by its non-toxic, non-immunogenic, and biocompatible nature, and it has received approval from the US FDA for use in certain medical devices [30]. Formulated with optimal rheological attributes, silk-based bioinks are suitable for precise 3D bioprinting, showcasing remarkable mechanical properties that facilitate the layer-by-layer construction of intricate tissue architectures and provide structural support akin to native tissues, mimicking their mechanical behavior. Additionally, silk-based hydrogels promote cell adhesion, proliferation, and differentiation within printed constructs, further enhancing tissue formation. The incorporation of bioactive agents, growth factors, or drugs into silk-based bioinks enables controlled and sustained release, which is advantageous for tissue regeneration and therapeutic applications. The inherent sol–gel property of silk protein’s polymer backbone, achieved through the physical crosslinking of β-sheet structures via hydrophobic interactions without involving chemical or photochemical crosslinking reactions [31], positions silk as a promising candidate for 3D bioinks due to its biocompatibility and ample mechanical strength for additive manufacturing. The degree of β-sheet physical crosslinking influences gel mechanical properties and in vivo degradation rates. Although numerous studies have explored silk proteins as bioinks for tissue engineering [32,33,34], some still employ chemical treatments, UV exposure, or ethanol [33] for gel formation. Furthermore, research addressing the tailoring of mechanical properties to meet specific organizational requirements remains limited [35].

**Figure 1:**
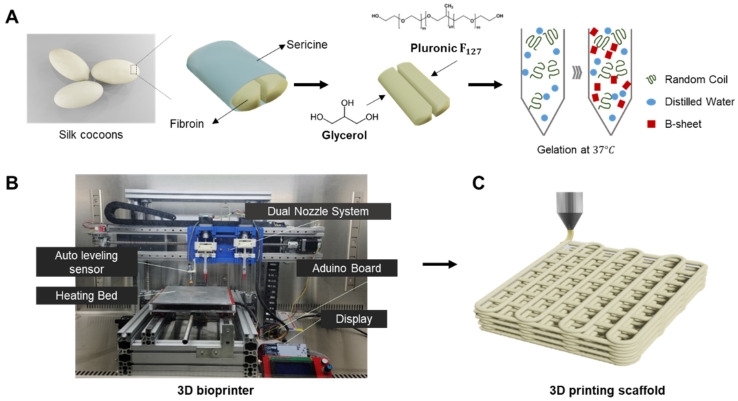
Schematic representation of this study. (**A**) Development of the silk fibroin-based bioink for 3D printing, involving modification of the physical properties of ink with different glycerol concentrations. (**B**) Extrusion-based 3D bioprinter. (**C**) 3D bioprinting with silk-based bioink.

Therefore, we propose to develop a hybrid bioink that exploits property complementarity by combining synthetic polymers and naturally derived polymers. In this study, we developed silk fibroin-based thermosensitive 3D printing bioinks (SF-TPBs) that can control the degree of β-sheet formation using a thermosensitive polymer and glycerol. Prior to this experiment, a screening test was performed to find the optimal mixing ratio of the materials used in the hydrogel. Consistent with our hypothesis, gelation was performed at 37 °C by treatment with F_127_, a thermosensitive polymer, and the gelation time decreased as the glycerol concentration increased. Thus, we have developed a bioink that can control gelation time and beta-sheet formation using a thermosensitive polymer and glycerol (Figure 1A). In addition, the physical properties, printability, and cytotoxicity of the glycerol hydrogels used as bioinks were systematically evaluated. These hybrid bioinks exhibit improved printability, mechanical strength, biocompatibility, and in vivo activity. SF-TPBs can be customized to meet the unique requirements of a desired tissue structure, including mechanical properties, biocompatibility, bioactivity, and desired cellular response [36].

## 2. Materials and Methods

### 2.1. Preparation of Silk-Based Bioinks

Silk fibroin from B. mori cocoons was kindly provided by the Rural Development Administration (Jeonju, Republic of Korea). Briefly, the sliced silk cocoons were subjected to two rounds of degumming using Marseilles soap (0.5% of fiber weight) and sodium carbonate solution (0.3% of fiber weight) at 100 °C for 1 h. Subsequently, they were washed with distilled water to eliminate silk sericin. The silk fibroin was hydrolyzed using 6 M Hydrochloric acid (HCl, 7647-01-0, Duksan Chemical, Ansan, Republic of Korea) for 5 h, with the reaction being neutralized using Sodium hydroxide (NaOH, 1310-73-2, Duksan Chemical, Republic of Korea). An electrodialysis system was employed to extract salt from the silk peptide solution. Upon salt removal, the silk fibroin was transformed into powder form through freeze-drying. The resultant silk peptide had an approximate molecular weight of 3000 Da [37]. Degummed silk fibroin and Pluronic F_127_ (Sigma-Aldrich, St. Louis, MO, USA) were dissolved in 10% (*w*/*v*) distilled water at 4 °C. Once the silk and F_127_ were completely dissolved, glycerol was added at various concentrations ranging from 15% to 55%. The resulting mixture was stored in a chamber at 37 °C until gelation occurred (Figure 1A).

### 2.2. Secondary Structure of Silk

The formation of the β-sheet structure was confirmed by Fourier transform infrared (FTIR) spectrophotometry. In this study, attenuated total reflection Fourier transform infrared (ATR-FTIR) measurements were performed using a SPECTRUM TWO Fourier transform infrared spectrometer (PERKIN ELMER, Waltham, MA, USA) equipped with a MIRacleTM attenuated total reflection germanium crystal. When infrared radiation is passed through a sample, the sample absorbs a portion of the radiation and transmits the remainder. The absorbed and transmitted energy can be measured, and the resulting signal is converted into a spectrum specific to the molecular composition of the sample. Background and spectral scans were obtained in the wave number range of 1000 to 2000 cm^−1^ with a resolution of 4 cm^−1^, with 32 scans per sample. To investigate the effect of glycerol treatment, peaks before and after gelation were measured, and any differences between the spectra were analyzed. This analysis provided insight into the changes in molecular structure, particularly the formation of β-sheet structures characteristic of silk fibroin.

### 2.3. Rheological Properties

Shear thinning is a rheological behavior exhibited by non-Newtonian fluids in which the viscosity of the fluid decreases as the shear rate increases. This behavior is often described as being similar to pseudoplasticity and is characterized by a lack of time dependence, such as thixotropy. In hydrogel printing, the shear thinning property plays a critical role in achieving accurate printing results. The hydrogel must maintain its fluidity while passing through the printing nozzle and then quickly recover its shape after extrusion. To quantitatively assess the gel and solution states of the hydrogel, rheological properties were measured. Rheological analysis of the bioinks was performed using an Advanced Rheometer AR 1500ex (TA Instruments, West Sussex, UK) equipped with a Peltier plate. The dynamic storage modulus (G’) and loss modulus (G”) of the materials were measured in oscillatory mode at a temperature of 37 °C. These measurements provide valuable information about the viscoelastic behavior of the material and its ability to undergo shear thinning and subsequent recovery.

### 2.4. Three-Dimensional Printing with a Silk-Based Thermosensitive Hydrogel

The 3D printer setup included essential components such as a controller board, stepper motor driver, LCD controller, and sensors. These electronic devices communicated with various sensors and executed commands to control the actuators. Printer operation was performed using the Repeater Firmware Configuration Tool (version 1.0.3), which allowed easy adjustment of parameters such as print speed and ink extrusion to achieve desired settings. The design of all 3D-printed structures was created using the Inventor program and saved as stereolithography (STL) files. These files were then converted to G-code files using the Cura 15.04.6 slicing program, which generated the necessary coordinates and paths for the 3D printer nozzles during printing.

To evaluate the physical properties of silk-based inks with different glycerol concentrations (15–55%) for 3D printing, we fabricated 3D structures of different sizes. The printed structures were used to compare the actual dimensions, including height and width, with the intended design dimensions. The printing process involved preparing a mixture of silk and F127 dissolved in a 10% solution, which was loaded into a syringe. Each concentration of glycerol (15–55%) was added to a syringe of equal volume and mixed through a syringe mixing tube. The resulting hydrogel mixture was then allowed to gel and stored in a chamber at 37 °C. After gelation, the hydrogel-filled syringe was attached to the 3D printer head. Prior to the extrusion of the silk-based mixture, the heated bed temperature was set at 37 °C. In the context of printing, a 22-gauge needle, corresponding to an inner diameter range of 420 μm, was utilized. The printed layer demonstrated a height of 200 μm. Tailored to the unique attributes of each silk-based mixture, the dispensing feed rate was established at 100 mm/min, and the printing speed was maintained at 5 mm/s for inks containing 15 to 25% glycerol. In the case of inks featuring 35% glycerol, the dispensing feed rate was adjusted to 120 mm/min, and the printing speed was set to 7 mm/s. Similarly, for inks comprising 45–55% glycerol, the dispensing feed rate and printing speed were configured at 105 mm/min and 5 mm/s, respectively.

### 2.5. Cell Viability Assay

MC3T3-E1 cells were cultured in α-MEM supplemented with 10% fetal bovine serum (FBS, Corning Cellgro, Corning, NY, USA) and 1% penicillin-streptomycin (Fisher Scientific, Waltham, MA, USA) under a 5% CO_2_ atmosphere at 37 °C. In addition, we assessed cell viability using LIVE/DEAD assays performed on silk-based hydrogels. The LIVE/DEAD cytotoxicity test kit provides a two-color fluorescence-based assessment of cell viability by simultaneously determining the proportion of live and dead cells. This method relies on two probes: cellular esterase activity and plasma membrane integrity. It provides an alternative to traditional methods such as trypan blue exclusion and 51Cr release for assessing cell viability and cytotoxicity. Cell viability is determined based on the ubiquitous presence of esterase activity in living cells, which catalyzes the enzymatic conversion of the virtually non-fluorescent cell-permeant calcein AM to a fluorescent product. This fluorescence-based method is faster, less expensive, safer, and more sensitive in detecting cytotoxic events than other approaches. Live cells retain the polyanionic dye calcein and exhibit a strong and uniform green fluorescence. Conversely, dead cells with compromised membranes allow the entry of EthD-1, a dye that undergoes a 40-fold increase in fluorescence upon binding to nucleic acids. This results in bright red fluorescence in dead cells because EthD-1 is unable to penetrate the intact plasma membrane of living cells. For 3D bioprinting of silk-based hydrogels, MC3T3-E1 cells were incorporated at a concentration of 7 × 10^6^ cells. Cells were harvested at 90% confluence and resuspended in supplemented α-MEM for encapsulation. Following the bioprinting of the MC3T3-E1-incorporated silk-based hydrogel onto a tissue culture dish, a 3 mL volume of α-MEM was introduced for subsequent cultivation. After 1, 3, and 7 days of culture, 100–150 µL of optimized concentrations of LIVE/DEAD test reagents were added to the silk hydrogels to ensure complete submersion of all cells in the solution. After incubation for 1 h at room temperature, labeled cells were visualized using a fluorescence microscope. The viability was calculated as a percentage by dividing the number of live cells by the total number of cells after counting cells using image J in the acquired image.

### 2.6. Releasing Test and Degradation Behavior

One of the important functions of inks used for bioprinting is the sufficient diffusion of oxygen, nutrients, and waste during culture. Therefore, we analyzed the release properties of silk-based hydrogels using bovine serum albumin (BSA) and bicinchonic acid (BCA). The BCA quantification method utilizes the property that proteins can reduce copper ions (Cu 2+, Cu 1+). Copper ions reduced by the protein react with the BCA solution to form purple compounds, and their absorbance is measured. Briefly, 1 mL of the silk-based mixture was combined with a 0.2% BSA solution, followed by dispensing the resulting mixture into Eppendorf tubes. The tubes were then incubated at 37 °C to facilitate gelation. Upon the transition of the mixture from a solution to a gel state, PBS was applied to it. The sample was subsequently placed in a controlled environment at 37 °C, and the absorbance was measured at regular intervals. The measuring solution was prepared by mixing a 50:1 mixture of PBS and BCA with PBS poured 1:1 on the sample and incubating for 1 h.

The degradation properties of bioinks offer crucial insights into their transient in vivo degradation and modifications. The assessment of degradation properties aids in comprehending the temporal degradation of inks within the environment and contributes to the evaluation of the sustainability and potential environmental consequences of products based on bioink. Specific bioinks confer distinctive functionalities or fulfill specific roles. A comprehensive comprehension of degradation characteristics enables the anticipation and management of alterations in ink performance over time. Deterioration measurements were conducted by transferring 200 μL of the silk-based mixture into Eppendorf tubes and incubating them at 37 °C for one day. Following this, 200 μL of PBS at 37 °C was added to each gelated sample, and the weight of the silk-based composite was determined by gently removing the PBS on the specified day. The measured weight was then calculated based on the dry weight of the lyophilized silk composite. This procedure was replicated with five samples over a span of approximately three months, and the measured weights were then arithmetically averaged.

## 3. Results

### 3.1. Silk Secondary Structure

**Figure 2:**
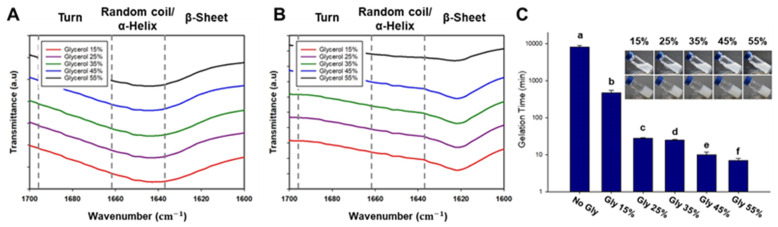
FTIR spectra of silk-based bioinks. (**A**) FTIR spectra of silk-based bioinks before gelation. (**B**) FTIR spectra of silk-based bioinks after gelation. (**C**) Gelation Time Assay. Determination of gelation time with varying glycerol concentrations (ANOVA, Duncan’s multiple range test, *p* < 0.05). Error bars indicate the standard error, and different letters indicate that the samples are statistically different.

Fourier transform infrared spectroscopy (FTIR) was used to investigate the structural characteristics of the printed silk-based hydrogel. The amide 1 region (1595–1705 cm^−1^) was analyzed, and specific peaks were assigned based on previous research [28,29,30,31]. According to a published paper, the peak at 1621 cm^−1^ is associated with the formation of β-sheet structures, while the peak at 1642 cm^−1^ corresponds to a random coil conformation. In the silk-based thermosensitive hydrogel with added glycerol, a peak at 1642 cm^−1^ was observed before gelation (Figure 2A). However, after gelation, a shift of the peak from 1642 cm^−1^ to 1621 cm^−1^ was observed, indicating the formation of β-sheet structures (Figure 2B).

To determine the gelation time of the silk-based ink treated with different concentrations of glycerol, the hydrogels were stored in a 37 °C chamber (Figure 2C). Gelation was considered to have occurred when the hydrogel did not flow when tilted. As expected, higher concentrations of glycerol resulted in shorter gelation times. Based on this observation, it was confirmed that glycerol reduces the time required for β-sheet structure formation. The gelation time for the groups treated with 25–45% (*v*/*v*) glycerol occurred within 30 min, while the group treated with 15% (*v*/*v*) glycerol exhibited a gelation time of 8 h.

### 3.2. Rheological Properties

The rheological properties of bioinks play a crucial role in 3D printing processes. The aim of this experiment was to analyze the rheological differences in silk-based thermosensitive hydrogels treated with different concentrations of glycerol. When the hydrogel is extruded under pressure through a small nozzle, it experiences shear stress. It should be in a solution state as it passes through the nozzle, and once extruded, it should retain the printed shape.

**Figure 3:**
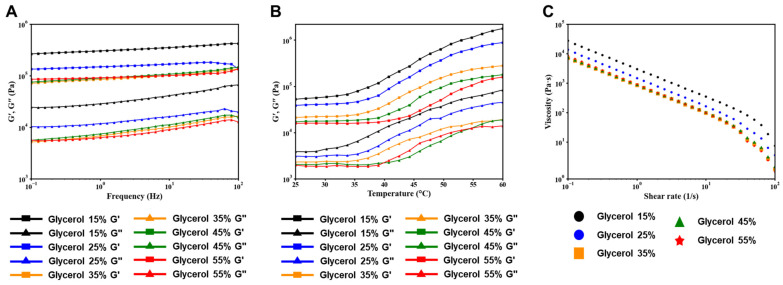
Rheological properties of silk-based bioinks with varying glycerol content. (**A**) Frequency-dependent storage modulus (G’) and loss modulus (G”) of the bioink formulations. (**B**) Temperature sensitivity of silk-based hydrogels. (**C**) Flow characteristics of different bioink formulations. Shear-thinning analysis according to each glycerol concentration.

Silk-based hydrogels treated with different concentrations of glycerol were prepared, and their rheological properties were evaluated (Figure 3A). Viscoelasticity was measured, and it was observed that, similar to most hydrogels, the storage coefficient (G’) was higher than the loss coefficient (G”). This indicates that the hydrogels exhibit predominantly gel-like behavior. And to demonstrate the temperature sensitivity of the hydrogel, we carried out a temperature sweep test (Figure 3B). The change occurred in the range between 35 °C and 60 °C. The group with glycerol content ranging from 15% to 35% exhibited an increase in viscosity around 37 °C. On the other hand, the group with a glycerol content of 45–55% exhibited relatively lower viscosity and showed an increase in viscosity at 45 °C. This phenomenon is attributed to the inverse relationship between glycerol content and the relative concentration of Pluronic F_127_, where higher glycerol content leads to a relatively reduced concentration of Pluronic F_127_. The silk-based hydrogels exhibited high viscosity and solid-like properties at low shear stress, and their shear thinning properties led to a decrease in viscosity with increasing shear rates (Figure 3C).

### 3.3. Printing Ability

**Figure 4:**
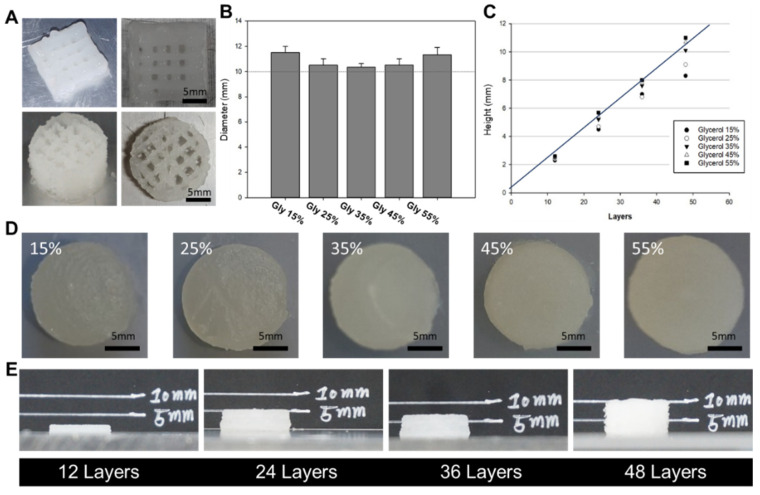
Evaluation of printing ability at different glycerol concentrations. (**A**) 3D printing in various scaffold forms. Printing of disk-shaped constructs: Printing of disk-shaped constructs with standardized dimensions (diameter: 15 mm, height: 10 mm). (**B**,**D**) Diameter analysis of 3D-printed outputs: Graph illustrating the diameter of 3D-printed outputs for each glycerol concentration. (**C**,**E**) Height analysis of 3D-printed outputs: Graph demonstrating the height of 3D-printed outputs for each glycerol concentration.

The size of the 3D-printed objects was measured using a caliper, and a comparison was made between the dimensions of the designed 3D objects and the actual printed output (Figure 4). The measurements showed a close resemblance between the modeling and the output, especially in the group treated with a glycerol concentration of 35% (*v*/*v*), which showed the highest resemblance. In the groups with glycerol concentrations below 35%, it was observed that the diameter of the printed output was larger, while the height was smaller than the designed dimensions. This phenomenon can be attributed to the ink extruding from the nozzle and spreading slightly sideways before complete solidification. In contrast, in groups with glycerol concentrations of 35% or higher, both the diameter and height of the printed output increased. This observation is likely due to residual stress within the syringe caused by the increased viscosity of the ink.

### 3.4. Cell Viability Assessment

As a preliminary experiment, we conducted a cytotoxicity evaluation of silk to assess its suitability as a bioink. Silk samples were treated with concentrations of 0%, 0.1%, 1%, and 10%. Cells were cultured in the presence of these silk samples, and cell activity was measured indirectly after 1 and 3 days using a WST-1 reagent to assess cell viability. The results of the toxicity assessment indicated that the groups treated with 0.1% and 1% silk showed higher cell activation than the control group after 1 day of culture. The group treated with 10% silk showed slightly lower levels of cell activation than the control group, although the difference was not statistically significant. After 3 days of evaluation, the silk-treated groups showed higher levels of cell activation than the control group, with the 1% silk-treated group showing the highest activation. After 7 days, the entire experimental group exhibited higher absorbance values than the control group, indicating increased cell activity. In conclusion, the cytotoxicity evaluation showed that silk, especially at concentrations of 0.1% and 1%, promoted cell activation and viability. These results suggest that silk has potential as a bioink material with favorable biocompatibility.

**Figure 5:**
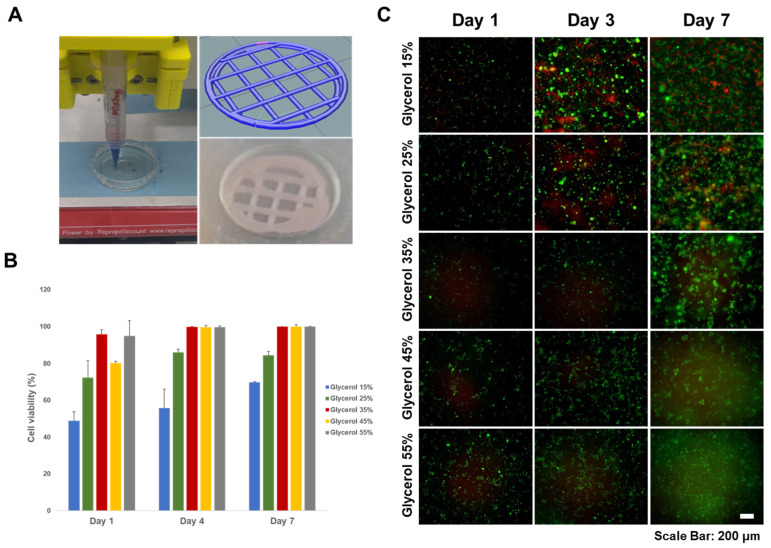
Cell proliferation analysis of 3D bioprinting hydrogels for each glycerol concentration. (**A**) 3D bioprinting with silk hydrogel. (**B**) Cell viability on days 1, 3, and 7 (ANOVA, Duncan’s multiple range test, *p* < 0.05). Error bars indicate the standard error. (**C**) Fluorescence microscopy images of LIVE/DEAD assay. MC3T3-E1 cultured in Z-stacked scaffolds after 1, 3, and 7 d. Green indicates live cells, and red indicates dead cells. The merged images indicate live and dead cells together.

We performed cell viability evaluations of silk-based hydrogels for use as bioinks in bioprinting applications (Figure 5). Among the different glycerol concentrations tested, the group treated with 15% glycerol showed the highest cell viability. However, the 15% glycerol-treated group tended to break easily due to low mechanical strength. This indicates incomplete gelation. On the other hand, the 25–35% glycerol treatment group showed relatively fast gelation, and cell viability was higher than that of the low glycerol treatment group. After 3 days, a few dead cells were observed, and there was general cell aggregation within the silk hydrogel. Cell viability increased over time, indicating that the silk hydrogels did not exhibit cytotoxic effects. In the 45–55% glycerol treatment group, cells did not survive and almost died. It is believed that this is the result of glycerol being dissolved and washed into the medium. Overall, the results suggest that silk-based hydrogels mixed with moderate amounts of glycerol are biocompatible and suitable for use as bioinks because they support cell viability and allow cell aggregation, which is desirable for tissue engineering applications. In conclusion, the evaluation confirmed no cytotoxicity in the silk hydrogel over time, indicating its potential as a safe and viable bioink material for bioprinting.

### 3.5. Releasing Test and Degradation Behavior

**Figure 6:**
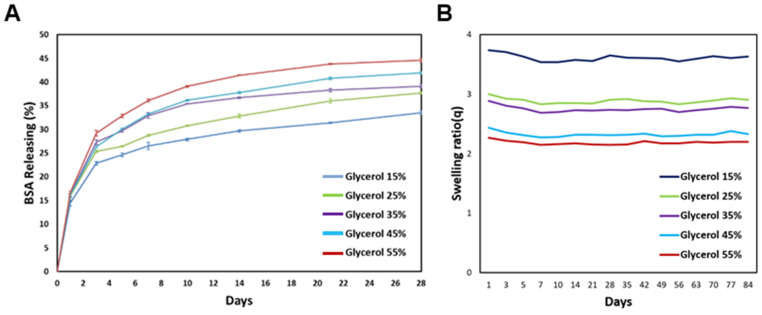
(**A**) Releasing test of hydrogel for 3D bioprinting by each glycerol concentration. (**B**) Degradation study of silk-based bioink.

In all experimental groups, there was a rapid release of BSA during the first week, followed by a gradual release over the next 20 days (Figure 6). Notably, the rate of BSA release was influenced by the glycerol concentration in the hydrogel. Higher glycerol concentrations resulted in faster BSA release, while lower glycerol concentrations exhibited relatively delayed release patterns. This observation may be attributed to the hydrophilic nature of glycerol. Hydrogels with higher glycerol concentrations have a greater affinity for the surrounding phosphate-buffered saline (PBS), resulting in more pronounced interactions and thus facilitating the faster release of BSA from the hydrogel matrix. These results indicate that the release properties of the silk-based hydrogel can be modulated by adjusting the glycerol concentration, thus providing control over the release kinetics of encapsulated bioactive molecules such as BSA. This knowledge is valuable for tailoring the delivery of bioactive substances in various biomedical applications.

PBS at 37 °C was applied to the silk-based composite gel samples, and the degradation behavior was assessed. The swelling ratio (Q), representing the ratio of the gel weight in the equilibrium-swollen state to the gel weight of the lyophilized silk-based composite, is depicted in Figure 6B. The silk-based gel sample exhibited a tendency to absorb a substantial amount of water on the first day, with lower glycerol content resulting in greater water absorption and subsequent weight gain. However, with the exception of the initial day, over the course of the 3-month experiment, the silk-based composite displayed no degradation, and no significant alterations in water content or weight were observed.

## 4. Discussion

Selecting an appropriate material is a critical step in using 3D printing technology to develop tissue regeneration constructs. The selected material must have several key characteristics. First, it should be printable, meaning it should have suitable viscoelastic properties that allow extrusion through a small nozzle and subsequent recovery without compromising its structural integrity. In addition, the material should be biocompatible, ensuring that it does not cause damage or adverse immune responses when in contact with living tissue. It is also essential that the material has the ability to encapsulate and support viable cells throughout the printing process. In the field of tissue engineering, the development of a hydrogel material that can be 3D-bioprinted to create scaffolds with uniform cell distribution has significant potential to promote improved tissue healing compared to conventional scaffolds. Silk fibroin (SF) is emerging as an ideal candidate for 3D bioprinting due to its unique properties. SF offers a controllable gelation process that allows precise control over scaffold formation. In addition, SF has a natural hydrogel structure that closely resembles the native extracellular matrix found in living tissues. By leveraging the controllable gelation and natural hydrogel properties of SF, it is possible to create 3D-printed constructs with tailored architecture and mechanical properties that provide an environment conducive to cell proliferation, differentiation, and tissue regeneration.

The gelation mechanism of silk fibroin (SF) has been extensively investigated, and it was found that SF, unlike other bioinks derived from natural materials, could undergo gelation through the formation of beta sheets. Remarkably, this gelation process did not require the use of chemical cross-linking methods. Silk protein is primarily composed of amino acids such as alanine (Ala), glycine (Gly), serine (Ser), and others. These amino acids form hydrogen bonds within the silk molecule, which is a crucial factor in determining the strength and stability of silk. Hydrogen bonds can take various forms within the silk molecule, and among them, the -OH (hydroxyl) groups play a significant role in hydrogen bond formation. Glycerol serves to evenly disperse silk within a hydrogel and plays a significant role in the hydrogen bond formation of silk-based hydrogels through its hydroxyl groups. These hydroxyl groups within the silk molecule induce interactions between protein chains by forming hydrogen bonds. Such interactions can induce gel formation. Hydrogen bonding between silk protein chains leads to the creation of a grid-like 3D structure, ultimately forming the solid structure of the gel. Leveraging the OH groups within silk molecules to form hydrogen bonds and induce gelation can lead to the development of materials that mimic physiological conditions, enhancing interactions with living systems. The rate of gelation could be controlled by adjusting the concentration of glycerol in the SF solution. By modulating the glycerol concentration, the rate of beta-sheet formation and, consequently, the gelation process could be controlled. Furthermore, when evaluating the rheological properties, which are crucial for determining the printability of hydrogels during the 3D printing process, the SF-based hydrogels exhibited shear-thinning behavior [38,39]. This means that the viscosity of the hydrogel decreases with increasing shear rates. As the hydrogel is extruded through the print nozzle, the shear rate increases, resulting in a decrease in viscosity. This shear-thinning behavior allows the hydrogel to flow more easily, facilitating the deposition of continuous strands during the printing process. Overall, the ability to control the gelation rate and shear thinning behavior of SF-based hydrogels through the incorporation of glycerol provides valuable control over the printability and rheological properties of the bioink. This knowledge is essential for optimizing the 3D printing process and achieving the desired structural fidelity and mechanical properties in the printed constructs.

The cylindrical scaffold design was chosen as a model system to determine the optimal formulation for achieving high morphological fidelity in 3D printing. These optimal formulation parameters can be used to print larger and more complex designs, which will be investigated in future studies. In our laboratory, we proposed to develop hydrogels optimized for 3D printing by utilizing SF-based hydrogel formulations with well-defined gelation times suitable for surgical applications. To achieve this, the glycerol content in the base formulation was modified to create an ideal formulation for 3D printing, taking into account our understanding of the gelation mechanism of SF. The gelled SF-based bioink showed excellent printability, as indicated by the storage coefficient G’, which was higher than the loss coefficient G’’ in all areas. However, the group treated with 15% glycerol exhibited prolonged gelation time and insufficient hydrogel viscosity, resulting in lateral spreading beyond the designed cylinder diameter and poor printing precision. On the other hand, the group treated with 55% glycerol showed a significantly reduced gelation time. However, the hydrogel exhibited excessive viscosity, resulting in excessive extrusion of the hydrogel due to residual shear forces, which compromised the quality of the printed scaffold. The range of 25–45% glycerol showed reasonable printing accuracy, with good results also observed in terms of cytotoxicity. Therefore, the optimal formulation is considered to be a bio-ink containing 25–45% glycerol. Overall, the results highlight the importance of optimizing the glycerol concentration in SF-based hydrogel formulations to achieve suitable gelation times and viscosities for 3D printing. The bioink formulation with 25–45% glycerol is considered optimal, offering good printability and compatibility, making it a promising candidate for bioprinting applications.

The MC3T3-E1 cells were successfully incorporated into the optimal SF-TPB hydrogel formulation and 3D-printed as scaffolds with high morphological fidelity. The printability of the system was not affected by cell culture medium or cell suspension. The encapsulation of cells within the hydrogel matrix offers advantages for tissue healing by allowing uniform cell infiltration and avoiding post-fabrication seeding processes that can hinder tissue regeneration. The MC3T3 cell line has the potential to induce uniform tissue formation throughout the encapsulated scaffold. To assess the viability and proliferation of the encapsulated cells, immediate post-print viability was evaluated after incubation in a calcium bath. Proliferation of the encapsulated cells was confirmed in the optimal SF-TPBs formulation, indicating that the printing process was not harmful to the cells and that the hydrogel formulation provided effective cell protection. However, the group treated with 55% glycerol showed low cell proliferation, which is consistent with literature reports suggesting that excessive glycerol can lead to cell death. However, as cell viability remains relatively high in other groups, the surviving cells retain the potential for recovery and proliferation through further in vitro culture, which will be investigated in future studies. In conclusion, the incorporation of appropriate glycerol concentrations not only increases the viscosity of the hydrogel formulation, but also indirectly improves cell viability after printing. The optimal SF-TPB formulation demonstrates the ability to support cell proliferation within the 3D-printed scaffolds, indicating its potential to promote tissue regeneration. This highlights the suitability of SF as a material for 3D bioprinting applications in tissue engineering and underscores its potential to advance the field of regenerative medicine.

## 5. Conclusions

SF-TPBs were developed using glycerol, and the rapid gelation and physical properties of silk gel during printing were modified to enable additive manufacturing. The silk/glycerol bioink was compatible with ABMSC in the gel matrix and supported excellent cell growth in gels at a concentration of 15–45% glycerol, but the environment in which cells lived in the group treated with 55% concentrations of glycerol was not supported. The SF-TPBs were gelled within a few minutes in the group treated with 25–55% concentrations of glycerol and have excellent 3D printing ability by using shear thinning properties. It can provide a silk-based gel system as a new bioink for 3D bioprinting.

## Data Availability

The data presented in this study are available on request from the corresponding author.
